# MeCP2 prevents age‐associated cognitive decline via restoring synaptic plasticity in a senescence‐accelerated mouse model

**DOI:** 10.1111/acel.13451

**Published:** 2021-08-07

**Authors:** Jin‐Lan Huang, Fan Zhang, Min Su, Jiaxin Li, Wen Yi, Li‐Xiang Hou, Si‐Man Yang, Jin‐Yuan Liu, Hao‐An Zhang, Tengfei Ma, Deng‐Pan Wu

**Affiliations:** ^1^ Jiangsu Key Laboratory of New Drug Research and Clinical Pharmacy Pharmacy School of Xuzhou Medical University Xuzhou China; ^2^ Scientific research center of traditional Chinese medicine Guangxi University of Chinese Medicine Nanning China; ^3^ Institute for Stem Cell and Neural Regeneration School of Pharmacy Nanjing Medical University Nanjing China; ^4^ Key Laboratory of Cardiovascular & Cerebrovascular Medicine School of Pharmacy Nanjing Medical University Nanjing China

**Keywords:** MeCP2, neurodegenerative disease, synaptic plasticity

## Abstract

Age‐related cognitive decline in neurodegenerative diseases, such as Alzheimer's disease (AD), is associated with the deficits of synaptic plasticity. Therefore, exploring promising targets to enhance synaptic plasticity in neurodegenerative disorders is crucial. It has been demonstrated that methyl‐CpG binding protein 2 (MeCP2) plays a vital role in neuronal development and MeCP2 malfunction causes various neurodevelopmental disorders. However, the role of MeCP2 in neurodegenerative diseases has been less reported. In the study, we found that MeCP2 expression in the hippocampus was reduced in the hippocampus of senescence‐accelerated mice P8 (SAMP8) mice. Overexpression of hippocampal MeCP2 could elevate synaptic plasticity and cognitive function in SAMP8 mice, while knockdown of MeCP2 impaired synaptic plasticity and cognitive function in senescence accelerated‐resistant 1 (SAMR1) mice. MeCP2‐mediated regulation of synaptic plasticity may be associated with CREB1 pathway. These results suggest that MeCP2 plays a vital role in age‐related cognitive decline by regulating synaptic plasticity and indicate that MeCP2 may be promising targets for the treatment of age‐related cognitive decline in neurodegenerative diseases.

## Graphical abstract

Decreased hippocampal MeCP2 expression contributes to age‐related cognitive decline by impairing synaptic plasticity. Upregulation of hippocampal MeCP2 could prevent age‐related decline in cognitive function and synaptic plasticity. MeCP2‐mediated regulation of synaptic plasticity may be associated with CREB1 pathway.

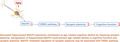

## 1 INTRODUCTION

Aging is a main risk factor for cognitive decline in neurodegenerative diseases, including various types of dementia. Despite extensive research on the etiology and acology of age‐associated cognitive decline in neurodegenerative diseases has been performed in the past decades, the effective therapy is limited(Partridge, 2014). It has been demonstrated that cognitive decline due to aging is associated with reduced synaptic plasticity in hippocampus(Bettio et al., 2017), prompting the suggestion that synaptic regulators might be promising targets to alleviate age‐related cognitive decline in neurodegenerative diseases.

Methyl‐CpG binding protein2 (MeCP2), a protein that is predominantly expressed in mature neurons, is believed to bind to methylated CpG dinucleotides, recruit chromatin remodeling proteins and corepressors to suppress gene expression (Kinde et al., 2016). Besides that, MeCP2 has been reported to function as gene activator(Horvath & Monteggia, 2018). There are reports showing that an approximate 85% of genes could be activated by MeCP2 in the hypothalamus of mice that either lack or overexpress MeCP2 (Chahrour et al., 2008). It has been demonstrated that MeCP2 plays a vital role in neuronal development and MeCP2 malfunction causes various neuronal disorders, such as Rett syndrome (RTT), neurobehavioral abnormalities, drug addiction, and MeCP2 duplication syndrome (Chin & Goh, 2019). However, the role of MeCP2 in neurodegenerative disorders has been less reported.

Previous studies have shown that the spine density of CA1 pyramidal cells of RTT patients was reduced in RTT patients and mice expressing RTT‐causing MeCP2 mutations(Chapleau et al., 2009). Additionally, alterations in MeCP2 expression have been shown to influence synaptic plasticity. Studies have reported that reductions in schaffer‐collateral long‐term potentiation (LTP) and long‐term depression (LTD) were observed in mice expressing disease‐causing *MeCP2* mutations and *MeCP2* null mice (Asaka et al., 2006; Weng et al., 2011). In contrast, hippocampal LTP responses were enhanced in MeCP2‐overexpressing mouse lines as compared to the controls (Collins et al., 2004). These findings indicate that MeCP2 holds the potential to regulate synaptic plasticity. In light of these reports, it is reasonable to hypothesize that MeCP2 might play a role in modulating synaptic plasticity in neurodegenerative disorders.

The senescence‐accelerated mouse prone 8 (SAMP8) is a well‐established age‐related mouse model presenting with spontaneous neurodegenerative changes associated with age‐related dementia, including age‐related learning and memory impairments and neuropathological features, such as neuronal loss, synaptic plasticity deficits, excessive Aβ generation, hyperphosphorylation of tau protein, neuroinflammation and oxidative stress, thereby considering as an appropriate model of age‐related neurodegenerative diseases (Liu et al., 2020; Morley et al., 2012; Pallas et al., 2008). Studies reported that 5‐month‐old SAMP8 showed significantly declined cognitive function and synaptic plasticity compared to age‐matched senescence accelerated‐resistant 1 (SAMR1) control strain (Lopez‐Ramos et al., 2012). Therefore, SAMP8 and SAMR1 mice were used to investigate the effect of MeCP2 on synaptic plasticity in neurodegenerative disease. The results illustrated that MeCP2 expression in hippocampus was reduced in mice upon senescence. Overexpression of hippocampal MeCP2 could elevate synaptic plasticity and cognitive function in SAMP8 mice. Oppositely, knockdown of MeCP2 could, to some extent, impair synaptic plasticity and cognitive function in SAMR1 mice. The work presents the ability of MeCP2 to enhance synaptic plasticity and indicates that strategies designed to maintain or elevate MeCP2 may be beneficial to the treatment of age‐related cognitive decline in neurodegenerative diseases.

## 2 RESULTS

### 2.1 MeCP2 expression is downregulated in the hippocampus of Alzheimer's disease patients and SAMP8 mice

**FIGURE 1:**
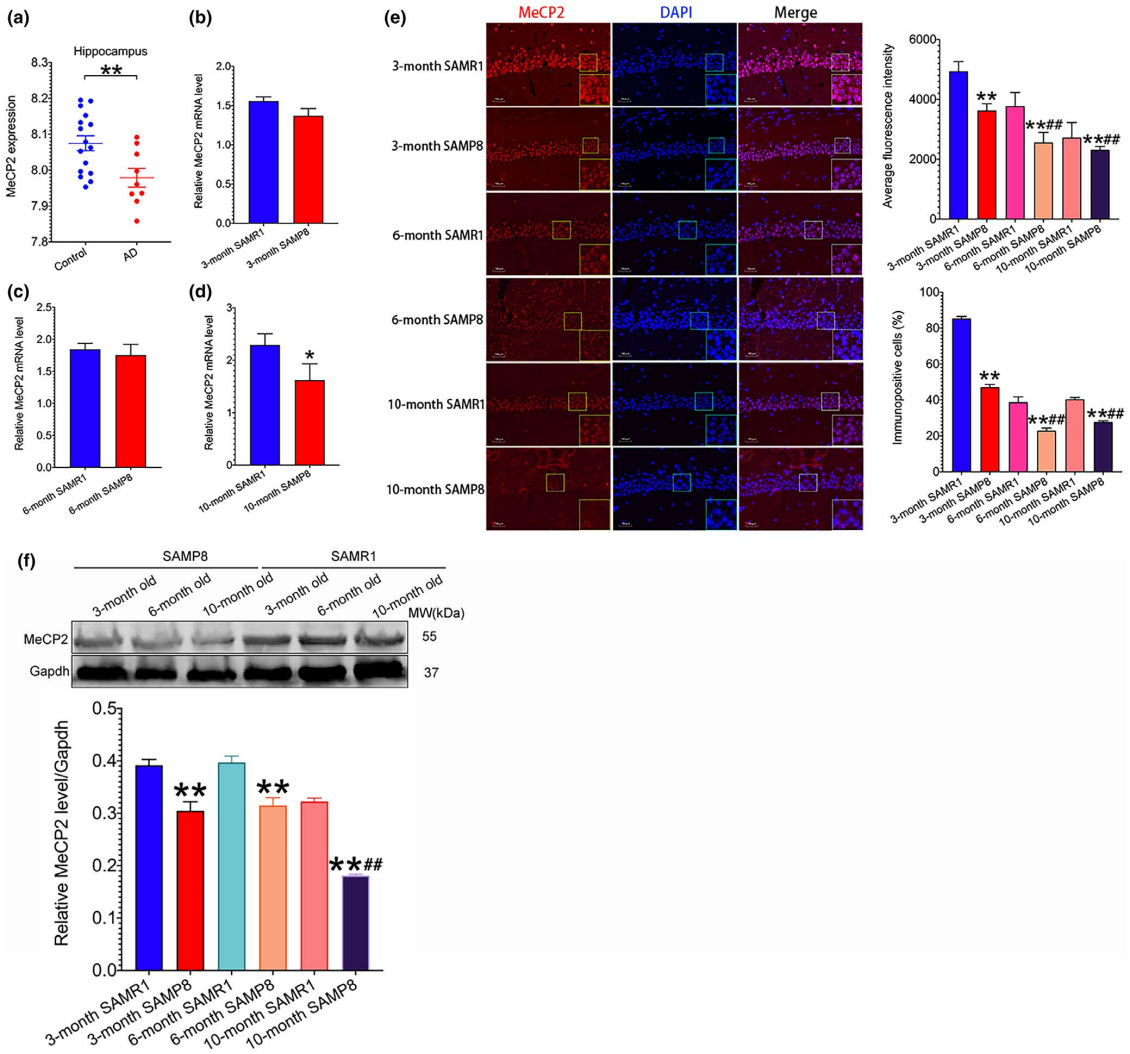
MeCP2 expression in AD patients and SAMP8 mice. (a) *MeCP2* mRNA expression of AD patients was analyzed. Data of *MeCP2* expression in hippocampus were form GSE29378 dataset in which 16 control and 9 AD subjects were included after data errors were corrected using Grubbs’ method. **p<0.01, versus control group; (b‐d) Hippocampal mRNA expression of *MeCP2* in SAMP8 mice with different months of age was detected using RT‐PCR assay. Data represent mean ±SEM. n=5 mice/group. *p<0.05, versus age‐matched SAMR1 mice; (e, f) MeCP2 protein expression was detected using IF and Western blot assay, respectively. Data represent mean ±SEM. n=3 mice/group. **p<0.01, versus age‐matched SAMR1 mice; ^##^ *p*<0.01, versus 3‐month‐old SAMP8 mice. Statistically significant differences were calculated by *t* test or two‐way ANOVA with Tukey's *post hoc* test using the SPSS 20.0 software

Since Alzheimer's disease (AD) is the most common form of dementia among age‐related neurodegenerative diseases, we first analyzed MeCP2 expression of AD patients in the GEO dataset (GSE29378) (data information was included in Table S1). The results revealed that the hippocampal expression of *MeCP2* was significantly downregulated after data errors were corrected using Grubbs’ method (Figure 1a). We then examined the hippocampal MeCP2 expression in SAMP8 mice with a different month of age. The results showed that *MeCP2* mRNA level was downregulated in the hippocampus of 10‐month‐old SAMP8 as compared to age‐matched SAMR1 (Figure 1b‐d). Additionally, the results of Western blot and immunofluorescence (IF) showed that the expression of hippocampal MeCP2 protein was decreased from 3‐ to 10‐month‐old SAMP8 compared with age‐matched SAMR1 (Figure 1e and f), indicating a post‐transcriptional regulation of MeCP2 expression.

### 2.2 Upregulation of MeCP2 improves cognitive function in SAMP8 mice

**FIGURE 2:**
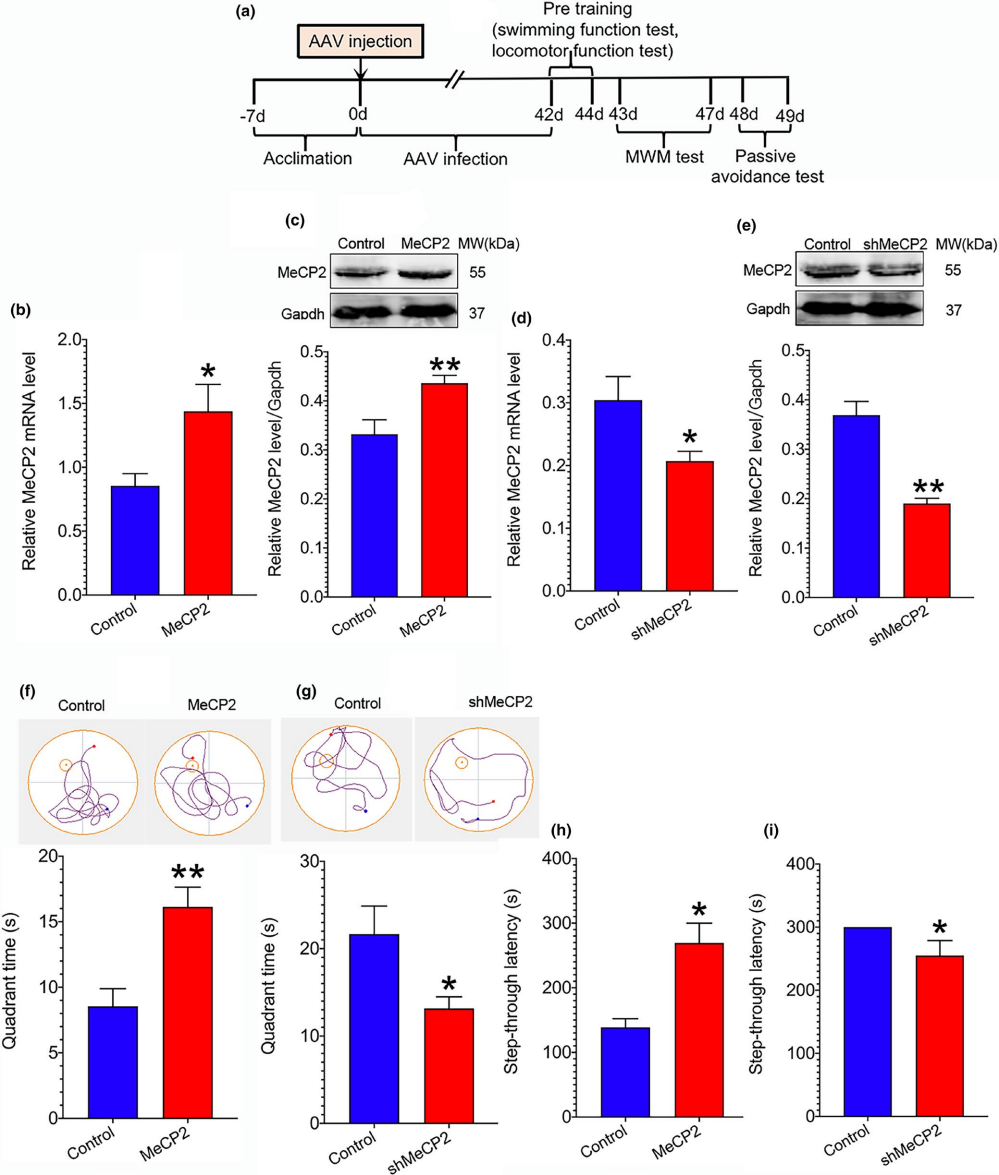
Effect of MeCP2 on cognitive function. AAV‐MeCP2 and AAV‐shMeCP2 particles and the corresponding AAV‐vector (control) were injected into the hippocampal region of SAMP8 and SAMR1, respectively, for 6 weeks. Then, MWM and light–dark passive avoidance tests were used to detect cognitive function of mice. (a) Illustration of the experimental procedure; (b, c) mRNA and protein expression of MeCP2 in SAMP8 mice. Data represent mean ±SEM. n=3 mice/group; (d, e) mRNA and protein expression of MeCP2 in SAMR1 mice. Data represent mean ±SEM. n=3 mice/group; (f, g) MWM test and light–dark passive avoidance test of SAMP8 mice, respectively. Data represent mean ±SEM. n=5 mice/group; (h, i) MWM test and light–dark passive avoidance test of SAMR1 mice, respectively. Data represent mean ±SEM. n=5 mice/group. Statistically significant differences were calculated by *t* test using SPSS 20.0 software. *p<0.05, **p<0.01, versus control group

We then investigated the role of MeCP2 in regulation of cognitive function in SAMP8 mice. For this purpose, AAV‐MeCP2 particles were injected into the hippocampal region to upregulate MeCP2 expression. As shown in Figure 2b and c, the mRNA and protein expression of MeCP2 was significantly elevated 6 weeks after AAV injection. The spatial learning and memory were measured using morris water maze (MWM) test. After training for four days, the time in the target quadrant of mice on day 5 was recorded. As demonstrated in Figure 2f, the quadrant time of mice treated with AAV‐MeCP2 was significantly prolonged as compared to the control mice, indicating that MeCP2 upregulation elevates the ability of learning and memory. Additionally, retention memory was detected using light–dark passive avoidance test after mice were subjected to 24h retention trial. As illustrated in Figure 2h, the step‐through latency was markedly prolonged in the mice overexpressing MeCP2 compared to the control mice, implying MeCP2 hold the potential to enhance retention memory. Collectively, the above results suggest that MeCP2 possess the capacity to improve cognitive function in senescence‐accelerated mice.

### 2.3 Downregulation of MeCP2 impairs cognitive function in SAMR1 mice

For further verifying the role of MeCP2 in cognitive function, we downregulated MeCP2 expression directly by microinjection of AAV‐shMeCP2 into hippocampal region to observe the change of cognitive function in SAMR1 mice. The expression of MeCP2 mRNA and protein was significantly reduced after mice were subjected to AAV injection for 6 weeks (Figure 2d and e). Cognitive function was evaluated using MWM and passive avoidance tests. As illustrated in figure 2g and i, mice pretreated with AAV‐shMeCP2 displayed significantly shortened quadrant time and step‐through latency as compared to the control mice, respectively, suggesting that downregulation of hippocampal MeCP2 impairs cognitive function.

### 2.4 Upregulation of MeCP2 enhances synaptic plasticity in SAMP8 mice

**FIGURE 3:**
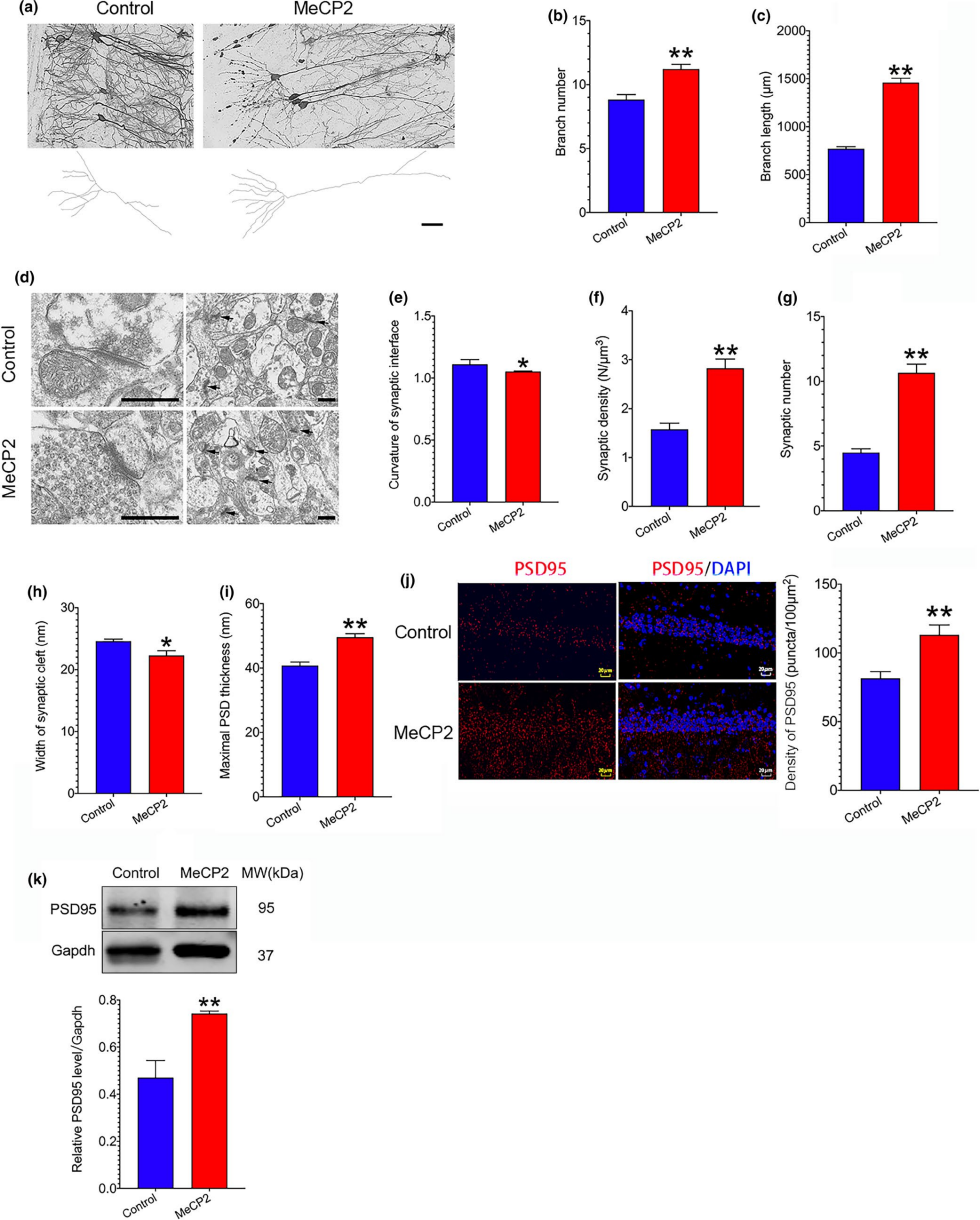
Effect of MeCP2 overexpression on synaptic plasticity in SAMP8 mice. 6 weeks after hippocampal injection of AAV‐MeCP2 and AAV‐vector (control), Golgi staining and TEM assays were performed. (a‐c) Structural synaptic plasticity changes of hippocampal neurons were observed using Golgi staining assay. Data represent mean ±SEM. n=3 mice/group. A total of 6~10 cells in each group were selected for data analysis; (d‐i) Structural synaptic plasticity changes of hippocampal neurons were measured using TEM assay. Data represent mean ±SEM. n=3 mice/group. A total of 6~11 cells in each group were selected for data analysis; (j) Neuronal synaptic marker PSD95 in mice overexpressing MeCP2 and control mice was detected by IF assay. The density of PSD95 puncta was analyzed using Image‐Pro Plus and ImageJ software. Data represent mean ±SEM. n=3 mice/group; (k) PSD95 protein expression was detected using Western blot assay. Data represent mean ±SEM. n=3 mice/group. Statistically significant differences were calculated by *t* test using SPSS 20.0 software. *p<0.05, **p<0.01, versus control group

Since synaptic plasticity is highly correlated with memory formation and retention, we further evaluated the role of MeCP2 in structural synaptic plasticity using Golgi staining and TEM assays. The results of Golgi staining revealed that the number of branches and branch length were significantly elevated in the hippocampus of mice treated with AAV‐MeCP2 as compared to the control mice (Figure 3a‐c). Additionally, the TEM technique was utilized to observe the ultrastructural alterations in the synapses of hippocampus. The results showed that compared with the control mice, the width of the synaptic cleft and curvature of the synaptic interface were remarkably decreased, whereas synaptic density, synaptic number, and maximal PSD thickness were significantly elevated in mice overexpressing MeCP2 (Figure 3d‐i). Furthermore, the expression of synapse‐associated protein PSD95 was detected using IF and Western blot assays. The result of IF assay showed that the density of PSD95 puncta in the hippocampal CA1 region of mice treated with AAV‐MeCP2 was significantly increased as compared to the control mice (Figure 3j). The result of Western blot assay revealed that PSD95 expression in MeCP2‐overexpressing group was remarkably elevated than that in the control group (Figure 3k). These results imply that upregulation of MeCP2 improves structural plasticity of synapses in SAMP8 mice.

It has been established that LTP, the prominent form of functional synaptic plasticity, has been reported to be the basis of learning and memory and impaired LTP has been correlated with cognitive deficits in dementing illness such as AD (Cuestas Torres & Cardenas, 2020; Kruijssen & Wierenga, 2019). We therefore investigated the effect of MeCP2 on LTP in acute hippocampal slices of SAMP8 mice overexpressing MeCP2. A standard HFS protocols were applied to induce LTP. As demonstrated in Figure 5a and b, the hippocampus slices of the mice overexpressing MeCP2 exhibited enhanced LTP as compared to the control mice. The validation of the peak amplitude of the fiber volley to the fEPSP slope and paired‐pulse facilitation (PPF) were shown in Figure S1a and c. Furthermore, under whole‐cell recording, we also found that the input–output curves of AMPAR‐EPSCs increased in MeCP2‐overexpressing group compared to the control group, while the PPF did not change between the control group and MeCP2‐overexpressing group, suggesting that overexpression of MeCP2 could enhance post‐glutamatergic transmission (Figure S2a and b). Lastly, we summarized the decay rate in MeCP2 and control groups. We found that there is no significant change of decay rate in both MeCP2 and control groups after HFS induction. But the decay rate in MeCP2 groups was higher than that in the control group (Figure S3a). HFS induced LTP in tetanized inputs in MeCP2 groups while the non‐tetanized synaptic input defined as control inputs was not found significantly changed, suggesting that the stability of glutamatergic transmission was recorded over the experimental time period (Figure S3b). Taken together, these data support the hypothesis that MeCP2 overexpression enhances synaptic plasticity in SAMP8 mice.

### 2.5 Downregulation of MeCP2 to some extent impairs synaptic plasticity in SAMR1 mice

**FIGURE 4:**
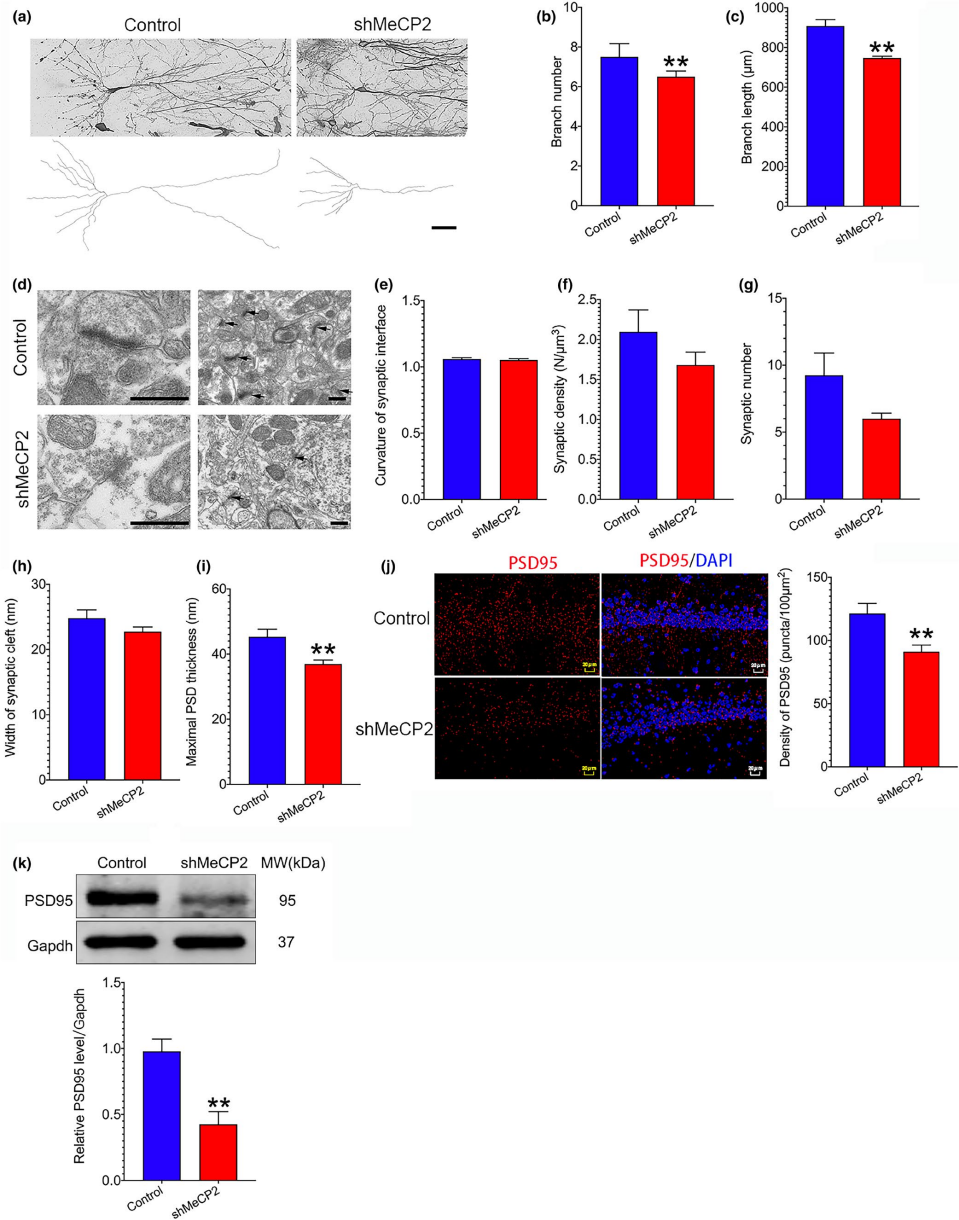
Effect of MeCP2 knockdown on synaptic plasticity in SAMR1 mice. 6 weeks after hippocampal injection of AAV‐shMeCP2 and AAV‐vector (control), Golgi staining and TEM assays were performed. (a–c) Structural synaptic plasticity changes of hippocampal neurons were observed by Golgi staining assay. Data represent mean ±SEM. n=3 mice/group. A total of 6~9 cells in each group were selected for data analysis; (d–i) Structural synaptic plasticity changes of hippocampal neurons were measured by TEM assay. Data represent mean ±SEM. n=3 mice/group. A total of 6~11 cells in each group were selected for data analysis; (j) Neuronal synaptic marker PSD95 in MeCP2 knockdown and control mice was detected by IF assay. The density of PSD95 puncta was analyzed using Image‐Pro Plus and ImageJ software. Data represent mean ±SEM. n=3 mice/group; (k) PSD95 protein expression was detected using Western blot assay. Data represent mean ±SEM. n=3 mice/group. Statistically significant differences were calculated by *t* test using SPSS 20.0 software. *p<0.05, **p<0.01 versus control group

**FIGURE 5:**
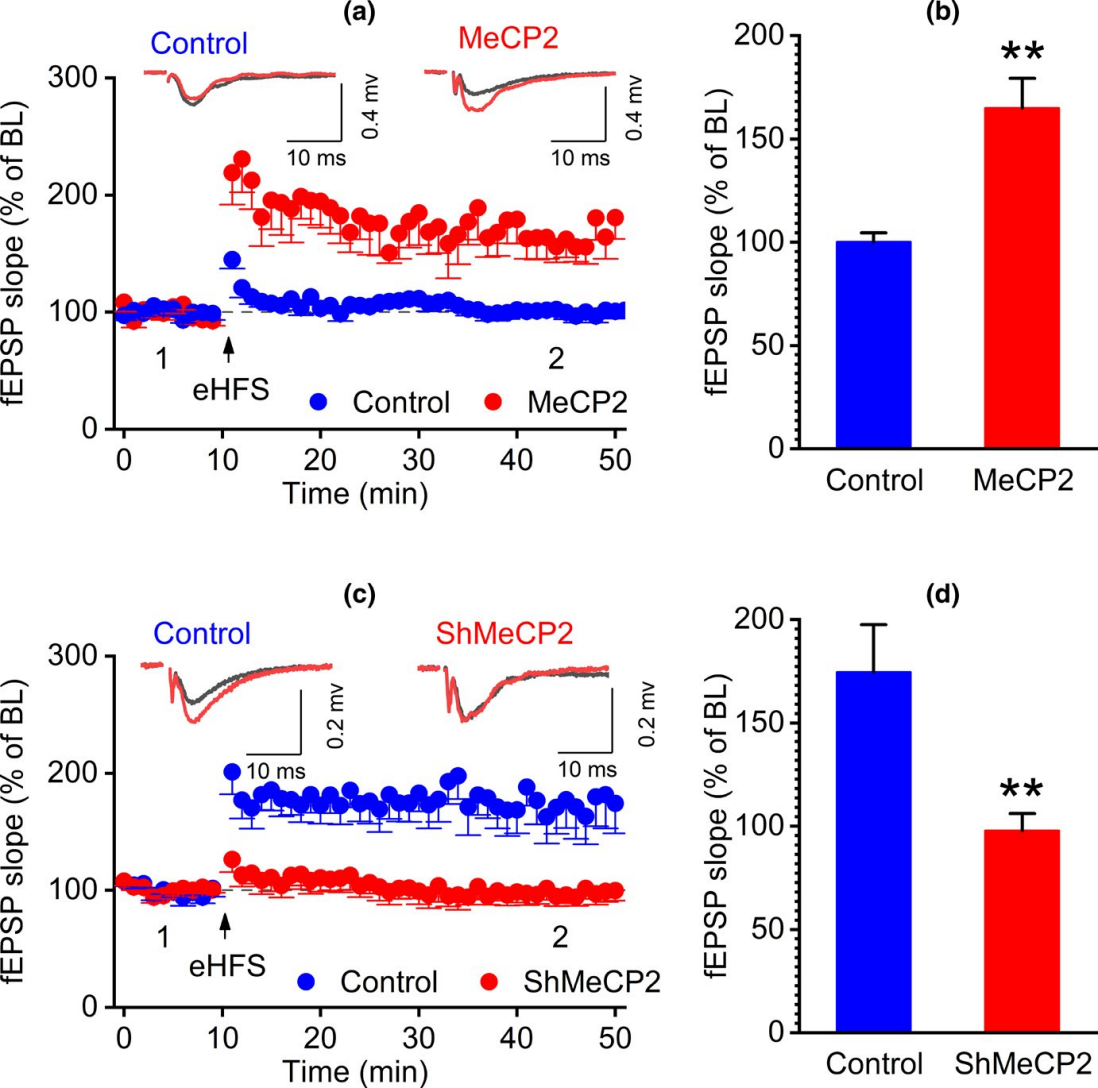
Effect of MeCP2 on LTP. 6 weeks after hippocampal injection of AAV‐MeCP2 and AAV‐shMeCP2 particles and the corresponding AAV‐vector (control), LTP of mice was detected. (a) eHFS induced robust LTP in slices of MeCP2 group: 164.89 ± 14.44% of BL, t(7) =4.494, *p* = 0.001; n = 8 slices, 4 mice; Control group: 100.17 ± 4.37% of BL, t(6) =0.040, *p* = 0.49; n = 7 slices, 4 mice. (Insets) Sample traces showing control and MeCP2 groups with a baseline period and 30–40 min period, respectively. Scale bars: 0.4 mV, 10 ms. (b) Summary of fEPSP slope in control and MeCP2 groups. t(13) =4.036, *p* = 0.001.(c) eHFS did not elicit LTP in slices of ShMeCP2 group mice (97.82 ± 8.28% of BL, t(6) = −0.26, *p* = 0.40; n = 7 slices, 4 mice). Control group: 174.58 ± 22.92% of BL, t(6) =3.25, *p* =0.008; n = 7 slices, 4 mice. (Insets) Sample traces showing control and shMeCP2 groups with a baseline period and 30–40 min period, respectively. Scale bars: 0.2 mV, 10 ms. (d) Summary of fEPSP slope in control and ShMeCP2 groups. t(12) =3.150, *p* = 0.008. Statistically significant differences were calculated by *t* test using SPSS 20.0 software. **p<0.01 versus control group

To further evaluate the role of MeCP2 in the plasticity of synapses of the hippocampus, we measured the structural and functional plasticity of synapses after hippocampal MeCP2 expression of SAMR1 mice was downregulated by hippocampal injection of AAV‐shMeCP2. As demonstrated in Figure 4a‐c, downregulation of MeCP2 led to reduced numbers of branch and branch length. Moreover, the maximal PSD thickness was significantly decreased in AAV‐shMeCP2‐injected mice compared to the control mice (Figure 4d and i). However, other indices such as synaptic density, synaptic number, width of synaptic cleft, and curvature of synaptic interface had decreasing trends, but had no statistical significance (Figure 4e‐h). Furthermore, the expression of synapse‐associated protein PSD95 was detected using IF and Western blot assays. The result of IF assay showed that the density of PSD95 puncta in the hippocampal CA1 region of mice treated with AAV‐shMeCP2 was significantly decreased as compared to the control mice (Figure 4j). The result of Western blot assay demonstrated that the expression of PSD95 in MeCP2‐knockdown group was remarkably reduced than that in the control group (Figure 4k). We also detected the effect of MeCP2 downregulation on hippocampal LTP. The results showed that MeCP2 downregulation resulted in a reduced hippocampal LTP (Figure 5c and d). The validation of the peak amplitude of the fiber volley to the fEPSP slope and PPF were shown in Figure S1b and d. In addition, the input–output curves of AMPAR‐EPSCs decreased in shMeCP2 group compared to the control group, while the PPF did not change between control and shMeCP2 groups, suggesting that post‐glutamatergic transmission was decreased by downregulation of MeCP2 (Figure S2c and d). These results suggest that downregulation of MeCP2 to some extent impairs plasticity of synapses in SAMR1 mice.

### 2.6 MeCP2‐mediated regulation of synaptic plasticity may be associated with CREB1 pathway

**FIGURE 6:**
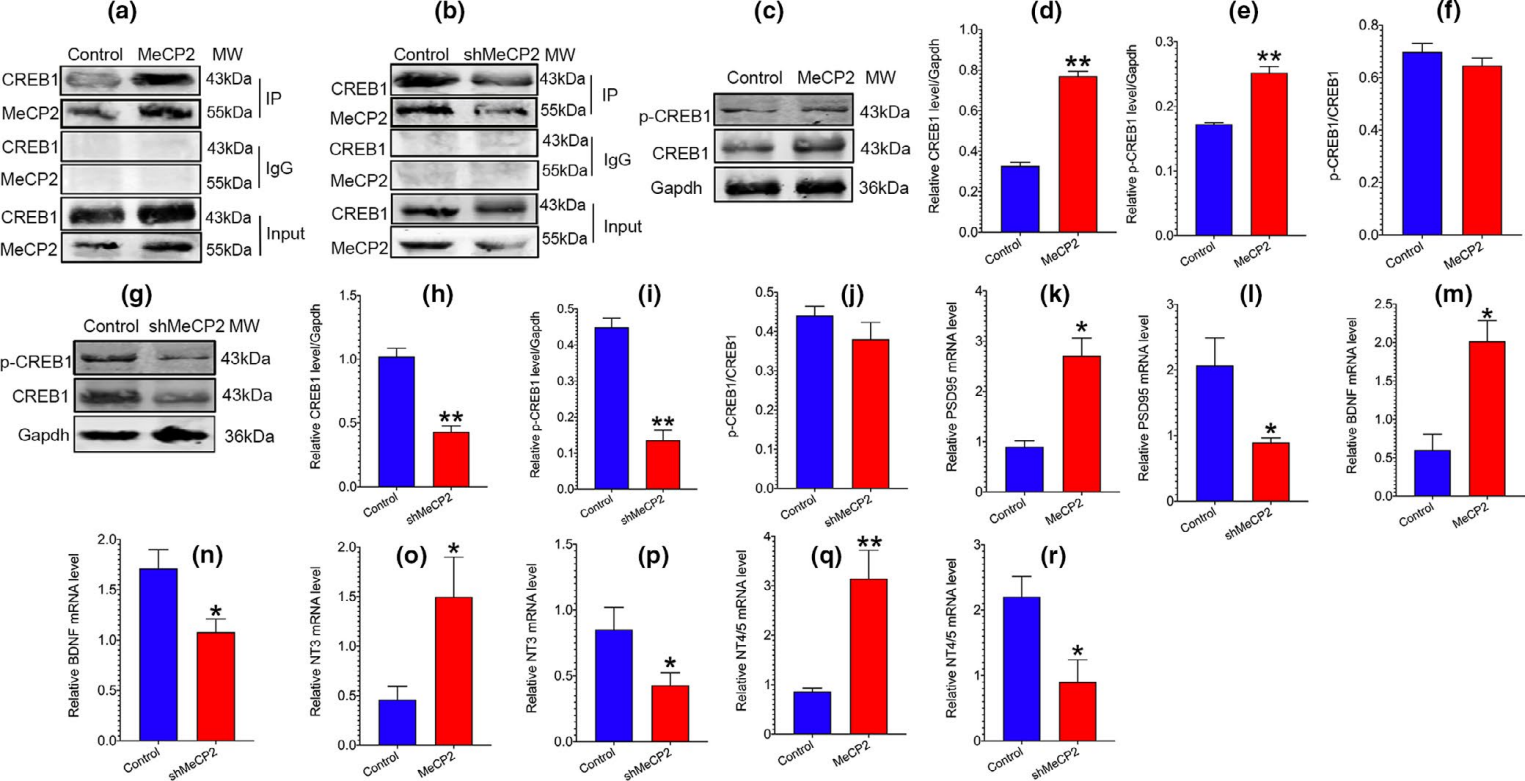
Effect of MeCP2 on CREB1‐mediated pathway. AAV‐MeCP2 and AAV‐shMeCP2 particles and the corresponding AAV‐vector (control) were injected into hippocampal region of SAMP8 and SAMR1 for 6 weeks, respectively. (a,b) The interaction of CREB1 with MeCP2 was detected by Co‐IP assay using MeCP2 antibody. (c‐f) The levels of total CREB1and p‐CREB1 (ser133) in SAMP8 mice overexpressing MeCP2 were detected using Western blot assay. (g‐j) The levels of total CREB1and p‐CREB1 (ser133) in MeCP2‐knockdown SAMR1 mice were detected using Western blot assay. For a‐j, data represent mean ±SEM. n=3 mice/group. (k‐r) The mRNA expression of *BDNF*, *PSD95*, *NT3*, *and NT4*/*5* in SAMR1 and SAMP8 mice was detected by RT‐PCR assay. Data represent mean ±SEM. n=4 mice/group. Statistically significant differences were calculated by *t* test using SPSS 20.0 software. *p<0.05, **p<0.01, versus control group

It has been documented that MeCP2 causes more gene activation rather than repression in the brain (Chahrour et al., 2008; Horvath & Monteggia, 2018). We assumed that MeCP2 might be associated with coactivators regulating synaptic plasticity. We then analyzed proteins interacting with MeCP2 using STRING (https://string‐db.org/cgi/input.pl) (Szklarczyk et al., 2019) and BioGRID (http://thebiogrid.org/) (Oughtred et al., 2019) online databases. We found that CREB1 had a high‐confidence interaction with MeCP2. CREB1 is a transcriptional activator that facilitates neuronal plasticity and long‐term memory formation, and has been reported to participate in regulating synaptic plasticity (Bartolotti & Lazarov, 2019). Thus, we hypothesized that CREB1‐mediated pathway might be associated with MeCP2‐mediated regulation of synaptic plasticity. We performed reciprocal immunoprecipitation with anti‐MeCP2 from hippocampal tissues after hippocampal MeCP2 expression was artificially altered. The results showed that MeCP2 could interact with CREB1 (Figure 6a and b). Additionally, we found that exogenously overexpressing MeCP2 could elevate the levels of CREB1 and p‐CREB1 (Figure 6c‐e). In contrast, downregulation of MeCP2 decreased the levels of CREB1 and p‐CREB1 (Figure 6g‐i). For verifying the role of MeCP2 in the modulation of CREB1‐mediated signaling, the expression of downstream synaptic genes of CREB1 pathway, BDNF, PSD95, neurotrophin 3 (NT3), and neurotrophin 4/5 (NT4/5) was detected (Choi et al., 2019; Juhasz et al., 2011; Sen et al., 2017). The results revealed that MeCP2 overexpression could improve the expression of *BDNF*, *PSD95*, *NT3*, *and NT4*/*5*, while downregulation of MeCP2 suppressed *BDNF*, *PSD95*, *NT3*, *and NT4*/*5* expression (Figure 6k‐r), indicating that MeCP2‐mediated regulation of synaptic plasticity may be associated with CREB1 pathway.

## 3 DISCUSSION

Aging is a major risk of neurodegenerative diseases associated with cognitive deterioration. It has been well established that the deficiency of synaptic plasticity is implicated in cognitive loss in neurodegenerative diseases (Skaper et al., 2017). Therefore, it is critical to identify potential targets to enhance synaptic plasticity in these disorders. The current study demonstrates a critical role of MeCP2 in the modulation of synaptic plasticity in senescence‐accelerated SAMP8 mice. Since AD is the most common form of age‐related neurodegenerative disorders, we first analyzed the expression of *MeCP2* based on a GEO dataset GSE29378 and found that *MeCP2* expression was downregulated in the hippocampus of AD patients (Figure 1a). Additionally, decreased hippocampal MeCP2 expression was found in SAMP8 mice (Figure 1c‐f). Upregulation of MeCP2 expression in the hippocampus enhanced synaptic plasticity and cognitive function in SAMP8 mice (Figures 2 and 3). On the contrary, downregulating hippocampal MeCP2 expression impaired synaptic plasticity and cognitive function in SAMR1 mice (Figures 2 and 4). These results indicate that MeCP2 could serve as an attractive target for conquering synaptic plasticity deficits and suggest that therapeutic strategies designed to remain or elevate MeCP2 expression may be beneficial to the treatment of age‐related cognitive decline in neurodegenerative diseases.

In the present study, a decreased *MeCP2* mRNA level in the hippocampus of AD patients was found through analyzing GEO datasets (Figure 1a). Inconsistent with our finding, Manavalan, et al. found that MeCP2 expression was elevated in the hippocampus after analyzing 4 AD and control subjects using isobaric tags for relative and absolute quantitation (iTRAQ) technology (Manavalan et al., 2013). This difference may be explained by the limited cases and different methods used for MeCP2 detection in the study. Additionally, we found that MeCP2 expression was downregulated in aged SAMP8 mice (Figure 1b‐f), which is consistent with the study indicating that both mRNA and protein levels of MeCP2 were reduced in APP/PS1 mice compared to age‐matched control mice (Choi et al., 2019). It should be noted that the mRNA level of *MeCP2* was not altered, while the protein expression of MeCP2 was downregulated in 3‐ and 6‐month‐old SAMP8 mice (Figure 1b‐f), implying that MeCP2 expression may be regulated via post‐transcriptional mechanisms.

Previous studies have shown that MeCP2 affects structural and functional synaptic plasticity in MeCP2 overexpression and MeCP2‐null mice. For example, elevated density of dendritic spine and LTP were found in the hippocampal slices from MeCP2^TG^ mice, a mouse line overexpressing MeCP2 (Collins et al., 2004). In contrast, a significantly reduced spontaneous excitatory synaptic transmission and impairments in LTP were observed in hippocampal slices from MeCP2‐null mice and in cortical slices from mice expressing disease‐causing mutations (Asaka et al., 2006; Weng et al., 2011). Consistent with these findings, in SAMP8 mice, we found that overexpression of MeCP2 in the hippocampus could increase structural synaptic plasticity in the hippocampal neurons of SAMP8 mice, manifested by increased PSD95 expression, elevated numbers of branch, branch length, and maximal PSD thickness, and decreased width of synaptic cleft and curvature of synaptic interface (Figure 3a‐k). On the contrary, downregulation of MeCP2 reduced PSD95 expression, branch numbers, branch length, and maximal PSD thickness (Figure 4a‐k). Additionally, MeCP2 overexpression enhanced hippocampal LTP, while knockdown of MeCP2 expression impaired hippocampal LTP (Figure 5a‐d). These results indicate that MeCP2 holds potential to regulate the plasticity of synapses in SAMP8 mice.

To date, the underlying mechanisms by which MeCP2 regulates synaptic plasticity have been less reported. It has been demonstrated that MeCP2 acts more as gene activator instead of gene repressor via interacting with transcriptional activator (Horvath & Monteggia, 2018; Yasui et al., 2007). The results of bioinformatics analysis revealed that CREB1, a transcriptional activator that has been shown to modulate synaptic plasticity in neurodegenerative diseases, may interact with MeCP2. Co‐IP assay showed that MeCP2 could interact with CREB1 (Figure 6a and b). Additionally, we found that MeCP2 overexpression increased CREB1 and p‐CREB1 level, while the depletion of MeCP2 expression suppressed the levels of CREB1 and p‐CREB1 (Figure 6c‐e,g‐i). These results are consistent with previous study indicating that MeCP2 could improve CREB1 expression by binding to the promoter region of CREB1 (Chahrour et al., 2008). However, modifying MeCP2 expression did not affect the ratio of p‐CREB1 to CREB1 (Figure 6f and j), indicating that regulation of p‐CREB1 expression by MeCP2 may be via modifying CREB1 expression. We further detected the expression of downstream synaptic genes of CREB1 pathway, the results of which showed that MeCP2 overexpression caused the upregulation of *BDNF*, *PSD95*, *NT3*, *and NT4*/*5* expression, whereas knockdown of MeCP2 expression inhibited *BDNF*, *PSD95*, *NT3*, *and NT4*/*5* expression (Figure 6k‐r). It has been demonstrated that MeCP2 and CREB1 combination could co‐occupy the promoter region of CREB1‐downstream genes, leading to an increase in the expression of target genes (Chahrour et al., 2008). Thus, MeCP2 and CREB1 interaction may be implicated in the enhanced expression of target genes by MeCP2. Taken together, the results imply that MeCP2‐mediated regulation of synaptic plasticity may be associated with CREB1 pathway.

Some limitations have to be interpreted in our results. Firstly, in the study, a hippocampal injection technique was performed; however, viral vectors may affect other brain regions with the circulation of cerebrospinal fluid. Thus, the influence of MeCP2 in other brain regions should be considered. Secondly, it has been reported that MeCP2 deficiency leads to the dysfunction of glial cells, which causes neuronal dendritic abnormalities (Kahanovitch et al., 2019; Sharma et al., 2018). Therefore, the role of glial MeCP2 in synaptic plasticity should be investigated in future studies. Thirdly, studies have shown that RNA interference has nonspecific toxicity and off‐target effects (Bartoszewski & Sikorski, 2019; Seok et al., 2018); thus, nonspecific toxicity and off‐target effects of RNA interference should be considered.

In conclusion, the work reported here demonstrates that decreased hippocampal MeCP2 expression may contribute to age‐related cognitive decline by impairing synaptic plasticity in hippocampal neurons. MeCP2 may be a novel target for the development of innovative drugs for age‐related cognitive decline in neurodegenerative diseases.

## 4 MATERIALS AND METHODS

### 4.1 Animals

6‐ to 8‐month‐old pathogen‐ and virus‐free SAMP8 and SAMR1 mice (Experimental animal center, Peking University) were housed in SPF conditions under a 12:12 h light/dark cycle at 25℃ and 45±5% humidity. Animal care and experimental procedures were implemented in accordance with the Institutional Animal Care and Use Committee of Xuzhou Medical University.

### 4.2 AAV construction and injection in mouse hippocampus

AAV construction was performed as previously described (Zhang et al., 2019). Briefly, pHBAAV‐CMV‐MCS‐3flag‐T2A‐ZsGreen and pHBAAV‐U6‐EGFP vectors were used to construct AAV‐MeCP2 and pAAV‐MeCP2‐shRNA (AAV‐shMeCP2, target sequence 5’‐CCGTGAAGGAGTCTTCCAT‐3’), respectively. 293T cells were transfected with pAAV‐RC, pHelper, and pHBAAV vectors. 72h after transfection, virus was collected from the cell lysate and purified using a heparin‐agarose column. Then, virus was concentrated and stored at −80℃ before use.

With regard to hippocampal injection of AAV virus, after anesthesia with 1% pentobarbital sodium, mice were fixed and the operative sites were sterilized. Using a 5‐μL Hamilton syringe, the AAV particles (1μL for each lateral) were injected into bilateral hippocampus according to the following coordinates: 1.94 mm posterior to bregma, 1.5 mm lateral to the midline, and 1.75 mm below the dura. For each injection, the needle was left in place for ten additional minutes before it was slowly removed. SAMP8 and SAMR1 mice were injected with AAV‐vectors.

### 4.3 Morris water maze test

Morris water maze (MWM) test was used to detect the capacity for spatial learning and memory as previously described (Huang et al., 2019). In the place navigation test, four consecutive daily training trials were carried out. In each trial, the mice were trained to locate the hidden platform. On day 5, a spatial probe test was performed to measure the capacity of spatial memory, in which the mice were subjected to a water pool without the hidden platform. The time spent in the target platform quadrant was recorded.

### 4.4 Light–dark passive avoidance test

The memory retention of mice was measured using light–dark passive avoid test in accordance with our previous study (Huang et al., 2019). The mice were placed in the illuminated compartment of automated passive avoidance system. Once the apparatus was activated, the door was raised allowing the mice to enter the dark compartment, where the mice were subjected to 2 seconds foot‐shock (0.2mA) on training day. The step‐though latency was recorded 24 h after the training session.

### 4.5 Golgi staining

Golgi staining was performed using Golgi‐Cox OptimStain^TM^ kit in accordance with the manufacturer's instruction. In brief, sliced brain samples were fixed with formaldehyde, rehydrated, and embedded in paraffin. Sections of brain were stained with staining solution for 10 min at room temperature. The images were taken with microscopy (Olympus BX‐50, Japan). Number of branches and branch length of neurons in the hippocampus were analyzed using ImageJ software by an observer blinded to the experiments.

### 4.6 Transmission electron microscopy (TEM)

Hippocampal tissues were fixed in 2.5% glutaraldehyde, treated with 1.5% potassium ferrocyanide and 1% osmium tetroxide, dehydrated in a graded series of ethanol solutions, and then embedded in an epoxy resin. Ultrathin sections were prepared and stained with citric acid lead and uranylacetate. A G2 Spirit Bio Twin transmission electron microscope (HT7700, HITACHI, Tokyo, Japan) was used to acquire the images. Width of synaptic cleft, synaptic density, synaptic number, and maximal PSD thickness was calculated according to Guldner's method (Guldner & Ingham, 1980). Curvature of synaptic interface was qualified using Jones’ method (Jones & Devon, 1978).

### 4.7 Electrophysiology

#### 4.7.1 Slice preparation

The procedure was conducted as described previously (Ma et al., 2018; Wei et al., 2018). Adult mice were sacrificed, and 250‐μm coronal sections containing the hippocampus were prepared in an ice‐cold protective cutting solution containing the following (in mM): NaCl 40, sucrose 148.5, KCl 4.5, NaH2PO4 1.25, NaHCO3 25, CaCl2 0.5, MgCl2 7, glucose 10, sodium ascorbate 1, and sodium pyruvate 3, Myo‐Inositol 3, saturated with 95% O2 and 5% CO2. Slices were then incubated in a 1:1 mixture of cutting solution and external solution at 32℃ for 45 min. The external solution was composed of the following (in mM): NaCl 125, KCl 4.5, CaCl2 2.5, MgSO4 1.3, NaH2PO4 1.25, NaHCO3 25, sucrose 15, and glucose 15, saturated with 95% O2 and 5% CO2. Slices were maintained in an external solution at room temperature until use.

### 4.8 Field potential recordings

Field excitatory postsynaptic potentials (fEPSP) were recorded with glass patch pipettes filled with 1 M NaCl positioned in the stratum radiatum of CA 1 area. A concentric electrode was used to elicit fEPSP by stimulation of the Schaffer collateral fibers. The slope of the rising phase of the fEPSP was used as a measure of the strength of synaptic transmission. Stimulus‐response curves were performed before each experiment. Baseline stimulation was delivered at a frequency of 0.05 Hz using a current intensity that elicited a 30–40% of the maximal fEPSP on the basis of input–output curves between stimulation intensity and evoked responses. A stable 10‐min baseline recording was preceded before LTP induction. LTP was induced by a high‐frequency stimulation (eHFS) containing a train of 100 stimuli delivered over 1 s, repeated 3 times at 10 s intervals. LTP was quantified by comparing the mean fEPSP slope during the baseline period over 30‐ to 40‐min post‐tetanus period and calculating the percentage change from baseline. fEPSP was recorded using an Axon clamp 700B amplifier (Axon Instruments). In Figure 5; Figure S1 and 3, fEPSP was recorded using an Axon clamp 700B amplifier or using IPA‐2 integrated patch amplifier controlled with SutterPatch software (Sutter Instrument, Novato, CA, USA). The experiments were measured in the presence of bicuculline (10 μM) to block the GABAergic transmission. The relationship of the peak amplitude of the fiber volley to the fEPSP slope (input/output curves) was compared in different groups (Figure S1). Paired‐pulse facilitation (PPF) was defined as the percentage increase in the second evoked response relative to the first in the pair using a 50‐ms interval between pulses.

### 4.9 Whole‐cell recordings

In whole‐cell voltage recording, neurons were clamped at −70 mV. AMPAR‐EPSCs was recorded at a frequency of 0.05 Hz in the presence of bicuculline. To measure AMPAR‐EPSCs input–output curves, varying intensities of electrical stimulation were delivered through bipolar electrodes. Paired‐pulse facilitation (PPF) was assessed ranging from 50 ms to 1000 ms intervals between pulses. The data were recorded using IPA‐2 integrated patch amplifier controlled with SutterPatch software (Sutter Instrument, Novato, CA, USA).

### 4.10 Real‐time PCR

After extraction of mRNA and reverse transcription, samples were mixed with SYBR Green Master Mix and primers using the Roche 480 LightCycler detection system. The primer sequences were as follows: MeCP2 (104bp), sense (TAGAAGGCCCAGCACAAAGT), antisense (CAGGGAGGGATGCTCTGTAG); BDNF (150bp), sense (CGACGACATCACTGGCTGACAC), antisense (GAGGCTCCAAAGGCACTTGACTG); PSD95 (80bp), sense (GCTATGAGACGGTGACGCAGATG), antisense (GTTGGCACGGTCTTTGGTAGGC); ACTB (130bp) sense (GTGCTATGTTGCTCTAGACTTCG), antisense (ATGCCACAGGATTCCATACC); NT3 (134bp) sense (CGTCAGTACTTCTTCGAGACG), antisense (ACATAGGACTGCTTAGCCTTG); NT4/5 (83bp) sense (ATGACAAACACTGGAACTCTCA), antisense (GCCTACGAGTTTGTTGTTTTCT).

### 4.11 Western blot assay

After extraction and separation by SDS‐polyacrylamide gel electrophoresis in 10% Tris‐glycine gels, the protein samples were transferred to a nitrocellulose membrane. Primary antibodies including MeCP2 (Santa Cruz Biotechnology, Texas, USA) (dilution: 1:500), PSD95 (1:500) (ABclonal, Wuhan, China), CREB1 (Cell Signaling Technology, Inc., Danvers, USA) (dilution: 1:700), p‐CREB1 (ser133) (Cell Signaling Technology, Inc., Danvers, USA) (dilution: 1:700), and GAPDH (Proteintech Group, Inc, Wuhan China) (dilution: 1:10000) were incubated at 4℃ overnight. Subsequently, IRDye purified secondary antibody (dilution: 1:10000) was incubated. Immunopositive bands were visualized at Ex/Em=778nm/795nm.

### 4.12 Co‐Immunoprecipitation (Co‐IP) assay

Co‐IP assay was performed in accordance with our previous study(Huang et al., 2019). In brief, after the supernatants of brain samples were collected, the lysates were incubated with MeCP2 antibody (Santa Cruz Biotechnology, Texas, USA) and protein A/G agarose gel overnight, followed by SDS‐PAGE resolution and Western blot assay. CREB1 antibody (Cell Signaling Technology, Inc., Danvers, USA) was used to measure the interaction of the two proteins.

### 4.13 Immunofluorescence

The paraffin‐embedded sections were rehydrated using graded alcohols. After antigen retrieval buffer and autofluorescence eliminator reagent (Beyotime Biotechnology, Shanghai, China) were added, MeCP2 antibody (dilution: 1:50) (Santa Cruz Biotechnology, Texas, USA), PSD95 (dilution: 1:100) (ABclonal, Wuhan, China), and secondary antibody included either goat anti‐rabbit Alexa Fluor 594 (ABclonal, Wuhan, China) were utilized. 4’,6‐diamidino‐2‐phenylindole (DAPI) (Beyotime Biotechnology, Shanghai, China) was used for nuclear staining. As for MeCP2, average fluorescence intensity was analyzed and immunopositive cells in each field were calculated using Image‐Pro Plus software (Media Cybernetics, Inc., USA). With respect to PSD95, the density of PSD95 puncta was analyzed using Image‐Pro Plus (Media Cybernetics, Inc., USA) and ImageJ software (Universal Imaging Corporation, USA) by an observer blinded to the experiments.

### 4.14 Statistical analysis

The data of each group were presented as mean±SEM. Grubbs’ method was used to correct data errors (Grubbs, 1969). *t* Test and two‐way ANOVA with Tukey's *post hoc* test were performed to analyze the variance using SPSS software for Windows 20.0. *p*<0.05 was considered to be statistically significant.

## CONFLICT OF INTEREST

The authors declare no conflict of interest.

## AUTHORS’ CONTRIBUTIONS

Deng‐Pan Wu designed the experiments. Jin‐Lan Huang, Fan Zhang, Min Su, Jiaxin Li, Li‐Xiang Hou, Wen Yi, Si‐Man Yang, Jin‐Yuan Liu, Hao‐An Zhang, and Tengfei Ma performed the experiments and analyzed the data. Deng‐Pan Wu wrote the paper. Jin‐Lan Huang, Fan Zhang, Min Su, Jiaxin Li, Li‐Xiang Hou, Wen Yi, Si‐Man Yang, Jin‐Yuan Liu, Hao‐An Zhang, and Tengfei Ma contributed analysis tools, reagents, and materials.

## Supporting information

Figure S1

Figure S2

Figure S3

Table S1

Figure Legends

## Data Availability

The processed data required to reproduce these findings cannot be shared at this time as the data also forms part of an ongoing study.
