# Supplementary material for: MeCP2 prevents age‐associated cognitive decline via restoring synaptic plasticity in a senescence‐accelerated mouse model
Source: Aging Cell. 2021 Aug 7;20(9):e13451. doi: 10.1111/acel.13451 (PMC8441320; doi:10.1111/acel.13451)
Supplement: Supplementary file 5 — Figure Legends [file ACEL-20-e13451-s004.docx]

**Supplementary Figure 1.** Validation of input-output relation and paired pulse facilitation (PPF) for synaptic plasticity in different groups. (a, b) Relationship of the peak amplitude of the fiber volley to the fEPSP slope (input/output curves) in Control and MeCP2 (a), and Control and ShMeCP2 mice (b). (c, d) PPF did not differ in MeCP2 (c), ShMeCP2 (d) with their controls.

**Supplementary Figure 2.** Input-output relation and PPF in MeCP2 and sh-MeCP2 groups and their control groups. (a) There is a significant difference in input–output curves for AMPAR EPSCs between MeCP2 group and control group. n = 23 in control group, n = 13 in MeCP2 group. Statistically significant differences were calculated by two-way repeated-measures ANOVA using SPSS 20.0 software. ##P<0.01, MeCP2 vs control. ** P<0.01, MeCP2 vs control in same intensity. (b) The inter-stimulus interval was set at 50-1000 ms. There is no significant difference in PPF between MeCP2 group and control group. n = 14 in control group, n = 16 in MeCP2 group. Statistically significant differences were calculated by unpaired t test using SPSS 20.0 software. (c) The input–output curves for AMPAR EPSCs in shMeCP2 group significantly decreased compared with control group. n = 15 in control group, n = 15 in shMeCP2 group. Statistically significant differences were calculated by two-way repeated-measures ANOVA using SPSS 20.0 software. ^#^*P*<0.05, shMeCP2 vs control. ** *P*<0.01, shMeCP2 vs control in same intensity. (d) There is no significant difference in PPF between shMeCP2 group and control group. n = 16 in control group, n = 15 in shMeCP2 group. Statistically significant differences were calculated by unpaired t test using SPSS 20.0 software.

**Supplementary Figure 3**. The stability of glutamatergic transmission over the recorded time period. (a) Summary of the decay rate at different time points in both MeCP2 groups and their control groups. The decay rate at 10 min (before HFS) was defined as baseline and the decay rate at 30 and 50 min was normalized to the decay rate at 10 min. In MeCP2 groups, the decay rate at 30 and 50 min was higher than at 10 min, respectively (^##^P< 0.01; ^###^P< 0.001). There is no significant change of decay rate between 30 min and 50 min. In control groups, there is no significant change of decay rate at 10 min, 30 min and 50 min. The decay rate in MeCP2 group was higher than in control group at 30 min (^*^P< 0.01) and 50 min (^**^P< 0.01), respectively. (b) The stability of glutamatergic transmission of control input was over the recorded time periods. The schaffer collateral was defined as tetanized LTP inputs and the performant path was defined as non-tetanized synaptic input (control inputs). The fEPSPs were recorded in the same neuronal population from tetanized input and non- tetanized input. The tetanized HFS of schaffer collateral input induced LTP (163.28 ± 20.85% of BL, t(5) = 3.04, p = 0.03) and non-tetanized inputs did not significantly change (106.96 ± 7.46% of BL, t(5) = 0.93, p = 0.39); n = 6 slices, 3 mice. Statistically significant differences were calculated by unpaired t test and paired t test using SPSS 20.0 software.
